# Morse subsets of CAT(0) spaces are strongly contracting

**DOI:** 10.1007/s10711-019-00457-x

**Published:** 2019-05-10

**Authors:** Christopher H. Cashen

**Affiliations:** Faculty of Mathematics, University of Vienna, 1090 Vienna, Austria

**Keywords:** Morse set, Contracting set, Recurrent set, CAT(0) space, Strongly contracting, 20F65, 20F67

## Abstract

We prove that Morse subsets of CAT(0) spaces are strongly contracting. This generalizes and simplifies a result of Sultan, who proved it for Morse quasi-geodesics. Our proof goes through the recurrence characterization of Morse subsets.

In this note we give a short proof of the following technical result:

## Proposition 1

If $\mathcal {Z}$ is a $\rho$-recurrent subset of a CAT(0) space $\mathcal {X}$ and the empty set is not in the image of the map $\pi _\mathcal {Z}(x):=\{z\in \mathcal {Z}\mid d(x,z)=d(x,\mathcal {Z})\}$ then $\mathcal {Z}$ is $12\rho (21)$–strongly contracting.

This is the final piece of the following theorem, which says that a number of properties that are equivalent to quasi-convexity in hyperbolic spaces are also equivalent to one another in CAT(0) spaces:

## Theorem 1

Let $\mathcal {X}$ be a geodesic metric space. Let $\mathcal {Z}$ be an unbounded subset of $\mathcal {X}$ such that the empty set is not in the image of $\pi _\mathcal {Z}$. The following are equivalent:

$$\mathcal {Z}$$**is Morse**
There is a function $\mu : [1,\infty )\times [0,\infty )\rightarrow [0,\infty )$ defined by $\mu (L,A):=\sup _\gamma \sup _{w\in \gamma } d(w,\mathcal {Z})$, where the first supremum is taken over (*L*, *A*)–quasi-geodesic segments $\gamma$ with both endpoints on $\mathcal {Z}$.
$$\mathcal {Z}$$**is contracting**
There is a function $\sigma : [0,\infty )\rightarrow [0,\infty )$ with $\lim _{r\rightarrow \infty }\sigma (r)/r=0$ defined by:
  $$\begin{aligned} \sigma (r):=\sup _{d(x,y)\le d(x,\mathcal {Z})\le r}{{\,\mathrm{diam}\,}}\pi _\mathcal {Z}(x)\cup \pi _Z(y) \end{aligned}$$
$$\mathcal {Z}$$**is recurrent**
There is a function $\rho : [1,\infty )\rightarrow [0,\infty )$ defined by:
  $$\begin{aligned} \rho (q):=\sup _{\varDelta (\gamma )\le q}\inf _{w\in \gamma } d(w,\mathcal {Z}') \end{aligned}$$
  The first supremum is taken over rectifiable segments $\gamma$ with distinct endpoints on $\mathcal {Z}$ such that $\varDelta (\gamma ):=\frac{{{\,\mathrm{len}\,}}(\gamma )}{d(\gamma ^+,\gamma ^-)}\le q$, where $\gamma ^+$ and $\gamma ^-$ are the endpoints of $\gamma$ and $\mathcal {Z}'$ is $\mathcal {Z}$ with the open balls of radius $d(\gamma ^+,\gamma ^-)/3$ about $\gamma ^+$ and $\gamma ^-$ removed.

If $\mathcal {X}$ is hyperbolic or CAT(0) then these conditions are equivalent to:

$\mathcal {Z}$**is strongly contracting**
$\mathcal {Z}$ is contracting and the contraction gauge $\sigma$ is a bounded function.

## Corollary 1

Morse subsets of CAT(0) spaces are strongly contracting.

We refer the reader to [3] for background on hyperbolic and CAT(0) spaces.

The Proposition and the Theorem can be extended to arbitrary non-empty subsets $\mathcal {Z}$ by suitable modification of the definitions. Specifically, if the empty set is in the image of $\pi _Z$ then redefine $\pi _\mathcal {Z}(x):=\{z\in \mathcal {Z}\mid d(x,z)\le d(x,\mathcal {Z})+1\}$. Extra bookkeeping is then required to compute an explicit contraction bound in the proof of the proposition. For bounded sets the four properties are trivially satisfied, with the possible exception that the given definition of recurrence does not make sense if some $\mathcal {Z}'$ is empty, which occurs, for instance, when $\mathcal {Z}$ is a two point set. We could redefine $\rho$ to be the diameter of $\mathcal {Z}$ in this case.

The corollary confirms a conjecture of Russell et al. [7] and generalizes a result of Sultan [8], who proved that Morse quasi-geodesics in CAT(0) spaces are strongly contracting.

Genevois [6] proved that Morse subsets of a finite dimensional CAT(0) cube complex $\mathcal {X}$ are strongly contracting *in the combinatorial metric*. While this is quasi-isometric to the CAT(0) metric, the property of being a strongly contracting subset is not, in general, preserved by quasi-isometries [2], so Genevois’s result and our theorem are independent. However, since the Morse property *is* preserved by quasi-isometries, and since Morse equals strongly contracting in both metrics, $\mathcal {X}$ has the same strongly contracting subsets regardless of whether it is endowed with the CAT(0) or the combinatorial metric.

## Proof of the theorem

The contraction condition was introduced in [1], where it was shown to be equivalent to the Morse condition. The recurrence condition was used to characterize Morse quasi-geodesics in [5], and this characterization can be extended to arbitrary subsets, as in [4, Theorem 2.2]. Strong contraction obviously implies contraction. It is easy to see that all of these properties are equivalent to quasi-convexity in hyperbolic spaces. The proposition supplies the remaining implication.$\square$

There is extensive literature making use of the Morse property and equivalent characterizations in various settings, but a complete exposition would be longer than this paper, so we will not attempt it. Sultan’s result uses a characterization of the images of Morse quasi-geodesics in asymptotic cones due to Druţu et al. [5]. Loosely speaking, this characterization depends on there being a sensible notion of one point being *between* two others, which we have for quasi-geodesics but not, at least in an obvious way, for arbitrary subsets. We avoid the use of asymptotic cones and instead use recurrence (which also comes from [5]). We construct curves in essentially the same way as Sultan, but our argument, in addition to applying to general subsets, is simpler and gives an explicit strong contraction bound.

## Proof of the proposition

Define $D:=\rho (21)$. Supposing the contraction gauge $\sigma$ of $\mathcal {Z}$ is not bounded by 12*D*, we derive a contradiction. Failure of the contraction bound means there exist points $x,\,y\in \mathcal {X}$ such that $d(x,y)\le d(x,\mathcal {Z})$ and such that ${{\,\mathrm{diam}\,}}\pi _\mathcal {Z}(x)\cup \pi _\mathcal {Z}(y)>12D$. We may assume $d(x,\mathcal {Z})\ge d(y,\mathcal {Z})$, because otherwise $d(x,y)\le d(y,\mathcal {Z})$ and we can swap the roles of *x* and *y*. Choose $x'\in \pi _\mathcal {Z}(x)$ and $y'\in \pi _\mathcal {Z}(y)$ such that $P:=d(x',y')>12D$. Let $\mathcal {Z}'$ denote the set $\mathcal {Z}$ with the open balls of radius *P* / 3 about $x'$ and $y'$ removed.

For points $a,\,b\in \mathcal {X}$, let $[a,b]: [0,1]\rightarrow \mathcal {X}$ denote the geodesic segment from *a* to *b*, parameterized proportional to arc length. Concatenation is denoted ‘$+$’.

* $$\begin{aligned} \text {If } d(w,\mathcal {Z}')\le D\hbox { for some }w\in \mathcal {X}\hbox { then }w\notin [x',x]+[x,y]+[y,y']. \end{aligned}$$

To see this, first suppose $w\in [x',x]$. Then $x'\in \pi _\mathcal {Z}(w)$, so $P/3\le d(x',\mathcal {Z}')\le d(x',w)+d(w,\mathcal {Z}')=d(w,\mathcal {Z})+d(w,\mathcal {Z}')\le 2d(w,\mathcal {Z}')\le 2D$, which is a contradiction, since $P>12D$. Similarly, $w\notin [y',y]$. If $w\in [x,y]$ then:

$$\begin{aligned} d(x,w)+d(w,y)=d(x,y)\le d(x,\mathcal {Z})\le d(x,w)+D \end{aligned}$$

Thus, $d(w,y)\le D$, which implies:

$$\begin{aligned} P/3\le d(y',\mathcal {Z}')\le d(y',y)+d(y,\mathcal {Z}')\le 2d(y,\mathcal {Z}')\le 2(d(y,w)+d(w,\mathcal {Z}'))\le 4D \end{aligned}$$

Again, this contradicts the hypothesis that $P>12D$, so (*) is verified.

Now there are three cases to consider.


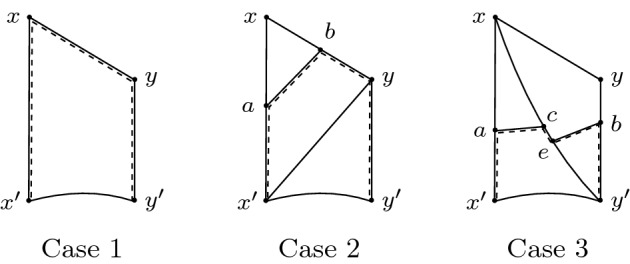


*Case* 1 $d(x,x')\le 6P$: Define $\gamma :=[x',x]+[x,y]+[y,y']$. Then ${{\,\mathrm{len}\,}}(\gamma )\le 18P<21P$, so recurrence says there is a point $w\in \gamma$ such that $d(w,\mathcal {Z}')\le D$. By (*), this is impossible.

*Case* 2 $d(x,x')>6P$ and $d(y,y')\le 4P$: Let $a:=[x',x](\frac{6P}{d(x,x')})$ and $b:=[y,x](\frac{6P}{d(x,x')})$, so that:

$$\begin{aligned} d(a,x')=\frac{6P}{d(x,x')}\cdot d(x,x') =6P \quad \text {and}\quad d(b,y)=\frac{6P}{d(x,x')}\cdot d(x,y)\le 6P \end{aligned}$$

Since $d(x',y)\le 5P$, the CAT(0) condition implies $d(a,b)< 5P$. Define $\gamma :=[x',a]+[a,b]+[b,y]+[y,y']$. Since ${{\,\mathrm{len}\,}}(\gamma )< 6P+5P+6P+4P=21P$, recurrence says there is a point $w\in \gamma$ with $d(w,\mathcal {Z}')\le D$. By (*), the only possibility is $w\in [a,b]$, but this is impossible because $d([a,b],\mathcal {Z})\ge d(a,\mathcal {Z})-d(a,b)> 6P-5P=P>D$.

*Case* 3 $d(x,x')>6P$ and $d(y,y')> 4P$: Let $a:= [x',x](\frac{4P}{d(x,x')})$ and let $c:=[y',x](\frac{4P}{d(x,x')})$. Then $d(x',a)=4P$ and:

$$\begin{aligned} 4P\le d(y',c)=\frac{4P}{d(x,x')}\cdot d(y',x)\le \frac{4P}{d(x,x')}\cdot (d(x,x')+P)< \frac{14}{3}P \end{aligned}$$

Let *b* be the point of $[y',y]$ at distance 4*P* from $y'$, and let *e* be the point of $[y',x]$ at distance 4*P* from $y'$, so $d(c,e)< \frac{2}{3}P$. The CAT(0) condition implies that $d(a,c)< P$ and, since $d(x,y)\le d(x,y')$, that $d(e,b)\le 4\sqrt{2}P$.

Define $\gamma :=[x',a]+[a,c]+[c,e]+[e,b]+[b,y']$. Then ${{\,\mathrm{len}\,}}(\gamma )< 4P+P+\frac{2}{3}P+4\sqrt{2}P+4P<21P$, so recurrence demands a point $w\in \gamma$ with $d(w,\mathcal {Z}')\le D$. By (*), $w\notin [x',a],\, [b,y']$. We cannot have $w\in [a,c]+[c,e]$ because $d([a,c]+[c,e],\mathcal {Z})\ge d(a,\mathcal {Z})-(d(a,c)+d(c,e))> 4P-P-\frac{2}{3}P>D$. Thus, $w\in [e,b]$, so $d(e,b)=d(e,w)+d(w,b)$. However, $d(w,b)\ge d(b,\mathcal {Z})-d(w,\mathcal {Z})\ge 4P-D>\frac{47}{12}P$. By the same reasoning, $\frac{47}{12}P<d(a,w)$, but $d(a,w)< P+\frac{2}{3}P+d(e,w)$, so $d(e,w)>\frac{27}{12}P$. This gives us the desired contradiction:

$$\begin{aligned} 6P<\frac{74}{12}P<d(e,w)+d(w,b)=d(e,b)\le 4\sqrt{2}P<6P \end{aligned}$$

Since all three cases ended in contradiction, we conclude 12*D* bounds $\sigma$.$\square$
